# Expression profile of genes associated with mastitis in dairy cattle

**DOI:** 10.1590/S1415-47572009005000074

**Published:** 2009-12-01

**Authors:** Isabela Fonseca, Priscila Vendramini Silva, Carla Christine Lange, Marta F. M. Guimarães, Mayara Morena Del Cambre Amaral Weller, Katiene Régia Silva Sousa, Paulo Sávio Lopes, José Domingos Guimarães, Simone E. F. Guimarães

**Affiliations:** 1 Laboratório de Genética Molecular, Embrapa Gado de Leite, Juiz de Fora, MGBrazil; 2 Departamento de Zootecnia, Universidade Federal de Viçosa, Viçosa, MGBrazil; 3 Departamento de Medicina Veterinária, Universidade Federal de Viçosa, Viçosa, MGBrazil

**Keywords:** cytokine, gene expression, immune response, real-time PCR

## Abstract

In order to characterize the expression of genes associated with immune response mechanisms to mastitis, we quantified the relative expression of the *IL-2*, *IL-4*, *IL-6*, *IL-8*, *IL-10*, *IFN-*γ and *TNF-* α genes in milk cells of healthy cows and cows with clinical mastitis. Total RNA was extracted from milk cells of six Black and White Holstein (BW) cows and six Gyr cows, including three animals with and three without mastitis per breed. Gene expression was analyzed by real-time PCR. *IL-10* gene expression was higher in the group of BW and Gyr cows with mastitis compared to animals free of infection from both breeds (p < 0.05). It was also higher in BW Holstein animals with clinical mastitis (p < 0.001), but it was not significant when Gyr cows with and without mastitis were compared (0.05 < p < 0.10). Among healthy cows, BW Holstein animals tended to present a higher expression of all genes studied, with a significant difference for the *IL-2* and *IFN-* γ genes (p < 0.001). For animals with mastitis no significant difference in gene expression was observed between the two breeds. These findings suggest that animals with mastitis develop a preferentially cell-mediated immune response. Further studies including larger samples are necessary to better characterize the gene expression profile in cows with mastitis.

## Introduction

Dairy cattle farming is one of the most important activities of the Brazilian agricultural industry; however, many obstacles still need to be overcome to ensure the sustainability and competitiveness of the sector. In regard to health problems, infectious-contagious diseases are the most important, being mastitis the main cause of economic losses. Mastitis is characterized by the presence of an inflammatory response in the mammary gland caused by metabolic and physiological alterations, injuries or, more frequently, environmental or contagious pathogenic microorganisms (Oviedo-Boyso *et al.*, 2007). Studies indicate that world economic losses due to mastitis can reach 35 billion dollars per year, with no actual statistics being available for Brazil, but it is believed that these numbers are significant (Polititis *et al.*, 1995; Giraudo *et al.*, 1997; Pereira *et al.*, 2001).

Zebu breeds and their crosses play an important role in the composition of Brazilian cattle, corresponding to about 80% of the national effective cattle herd. The Gyr breed plays a key role in this context and its importance has been increasing in Brazil and other countries in Latin America, as well as in Africa and Asia, because this breed is widely used in crossings, especially with Holstein animals, aiming to incorporate rusticity, productivity, docility, and its effectiveness in the production of milk at low cost (Ferreira *et al.*, 2007; Vercesi Filho *et al.*, 2007). In contrast, Holstein cattle is the most widely reared European breed in Brazil (Costa *et al.*, 2007) and it is known worldwide as the major milk producer among cattle breeds.

One promising approach to reduce problems caused by infectious-contagious diseases, in addition to sanitary care, is the selection of animals that are resistant to disease and the incorporation of this trait into the herds. In the case of mastitis, Detilleux *et al.* (1994) stated that it will be difficult to produce effective vaccines due to the wide variety of microorganisms causing the disease. Within this context, studies aiming for a better understanding of the biological processes involved in the determination of resistance to diseases are fundamental to overcome these problems and to develop technological solutions.

Resistance to mastitis is a complex trait and genes involved in the immune response have been indicated as strong candidates (Shuster *et al.*, 1993; Ferens *et al.*, 1998; Alluwaimi *et al.*, 2003; Rambeaud *et al.*, 2003; Oviedo-Boyso *et al.*, 2007;). Therefore, the objective of the present study was to characterize the expression of genes associated with immune response mechanisms to mastitis. For this purpose, the expression of the interleukin 2 (*IL-2*), *IL-4*, *IL-6*, *IL-8*, *IL-10*, interferon gamma (*IFN-*γ) and tumor necrosis factor alpha (*TNF-*α) genes was investigated in Black and White (BW) Holstein and Gyr cows with and without clinical signs of mastitis.

## Material and Methods

Six BW Holstein cows (*Bos taurus*) and six Gyr cows (*Bos indicus*) raised by two different commercial farms in the State of Minas Gerais, Brazil, were used. The animals selected from each breed were divided into two groups: 1) free of infection (n = 3) and 2) with clinical mastitis (n = 3).

A 150-mL aliquot of milk was collected from one quarter of each cow into sterile tubes. All animals were submitted to clinical examination of the udder before collection of the samples. The milk samples from cows with mastitis were collected from the quarter with clinical mastitis immediately after the onset of clinical signs and before drug treatment, thus excluding artificial infection of the cows. In addition, all samples were submitted to microbiological analysis.

Total RNA was extracted from the milk samples using the RNeasy Mini kit (Qiagen, Valencia, CA, USA) according to manufacturer instructions. The extracted RNA was quantified spectrophotometrically and the OD_260_/OD_280_ was used for evaluation of quality. First-strand cDNA was synthesized using the SuperScript III First-Strand Synthesis SuperMix kit (Invitrogen, Carlsbad, CA, USA) and the average concentration of cDNA in the samples was estimated by spectrophotometry. The cDNA samples were then stored at -20 °C until use in real-time PCR.

Real-time PCR was carried out using the SYBR Green® PCR Master Mix kit (BioRad, Hercules, CA, USA) according to manufacturer instructions. The primers used for the evaluation of gene expression were designed as described in the literature (Table 1). The glyceraldehyde-3-phosphate dehydrogenase (*GAPDH*) gene was used as endogenous reference (Leutenegger *et al.*, 2000). After 40 amplification cycles, all samples were submitted to analysis of the dissociation curve in order to confirm the absence of nonspecific products and primer dimers.

Before real-time quantification, the PCR was optimized for all genes. For this purpose, three cDNA concentrations (10, 100 and 200 ng/μL) and three primer dilutions (100, 200 and 400 nM) were tested. After determination of the best PCR conditions, a standard curve was constructed for each gene in which the serial cDNA dilutions were plotted against their respective cycle thresholds (Ct). The efficiency of amplification of the target genes and endogenous control was similar, ranging from 0.61 to 0.74, and the dissociation curves showed no peaks corresponding to primer dimers or nonspecific products for any of the target genes or endogenous control. Table 1 shows the cDNA and primer concentrations optimized for each gene. For the *IL-2* and *IL-6* genes, the best efficiency of the reactions was obtained when 400 ng cDNA/μL was used. The coefficient of variation in Ct obtained for each sample in duplicate reactions did not exceed 5%, with the widest variation being observed for the *IL-6* gene in one Gyr cow without mastitis (2.95%).

Each sample was analyzed in duplicate in 96-well optical reaction plates sealed with optical adhesive film. The samples were amplified separately using the ABI Prism 7300 Sequence Detection System (Applied Biosystem) by the amplification program (denaturation at 95 °C for 5 min, followed by 40 cycles with denaturation at 95 °C for 15 s, and annealing and extension at 60 °C for 1 min). The real-time PCR results were analyzed with the REST^©^ program (Pfaffl *et al.*, 2002), which uses the Pair Wise Fixed Reallocation Randomisation Test^©^ to compare differences in expression across treatments.

## Results

Microbiological analysis indicated *Streptococcus* spp. as the causative agent in the three BW Holstein cows with mastitis and *Klebsiella pneumonia* in one Gyr cow. Isolation in culture was not possible in the remaining cows due to the presence of multiple causative agents.

The expression of the *IL-2*, *IL-4*, *IL-6*, *IL-8*, *IL-10*, *IFN-*γ and *TNF-*α genes were compared in: I) animals without mastitis *vs.* animals with mastitis of both breeds (Table 2); II) BW Holstein cows without mastitis *vs.* BW Holstein cows with mastitis (Table 2); III) Gyr cows without mastitis *vs.* Gyr cows with mastitis (Table 2); IV) BW Holstein cows without mastitis *vs.* Gyr cows without mastitis (Table 2); V) BW Holstein cows with mastitis *vs.* Gyr cows with mastitis (Table 2).Considering the two breeds together (Table 2), *IL-10* expression was 20.71 times higher in cows with mastitis compared to animals without mastitis (p < 0.05). For the other genes, no significant increase or decrease in expression across groups were observed (p > 0.05).

Similarly, when analyzing only BW Holstein cows (Table 2), *IL-10* expression was also higher (35.66 times) in cows with mastitis compared to animals free of infection (p < 0.001), whereas no difference in expression between animals with and without intramammary infection was observed for the other genes studied (p > 0.05). In the case of Gyr cows (Table 2), no significant difference (p > 0.05) in *IL-10* gene expression was observed between cows with and without mastitis; however, *IL-10* expression showed a trend to be 12.03 times higher in cows with mastitis compared to healthy animals (p < 0.10).

Comparison of animals of the two breeds without mastitis (Table 2) showed a significantly lower expression of the *IL-2* and *IFN-*γ genes (p < 0.001) in Gyr cows, whereas no significant difference in expression between Gyr and BW Holstein cows was observed for the other genes (p > 0.05). For animals with mastitis (Table 2), no significant difference in the expression of any gene was observed between the two breeds (p > 0.05).

## Discussion

In the present study, higher expression of the *IL-10* gene was observed in BW Holstein and Gyr cows with mastitis compared to healthy animals, whereas the other studied genes presented no significant difference in expression. IL-10 is an anti-inflammatory cytokine able to inhibit natural killer cells (Barral-Neto *et al.*, 1995), as well as the production of IL-1 and TNF-α by macrophages (Fiorentino *et al.*, 1991), and also the production of IFN-γ and IL-2 by Th1 lymphocytes (Mosmann and Moore, 1991). This fact suggests that high levels of IL-10 may have suppressed the expression of *IL-2*, as well as the expression of *TNF-*α and *IFN-*γ. The present results agree with the findings of Riollet *et al.* (2001) who observed a higher expression of *IL-10* in milk cells of animals with chronic mastitis caused by *Staphylococcus aureus* compared to healthy cows. In addition, various studies have shown that *IL-10* is expressed in milk cells of udders infected with different pathogens (Bannerman *et al.*, 2004a, 2004b, 2005).

Similarly, separate analysis of BW Holstein cows also showed a higher expression of the *IL-10* gene in animals with mastitis compared to animals free of infection. However, analysis of Gyr cows revealed no significant difference in *IL-10* gene expression between animals with and without mastitis, although *IL-10* expression showed a trend to be higher in cows with mastitis compared to healthy animals (p < 0.10). These results suggest a higher expression of the *IL-10* gene in BW Holstein cows with mastitis when compared to Gyr cows with mastitis. In fact, as can be seen in Table 2, expression of this gene tended to be 7.35 times lower in Gyr cows with mastitis when compared to BW Holstein cows with mastitis, but this difference was not significant (p > 0.05). In addition, the significant difference in gene expression between animals of both breeds with and without mastitis (Table 2) was probably due to the large difference in expression between healthy animals and BW Holstein cows with mastitis (Table 2).

For Gyr cows (Table 2), expression of the *IL-4* gene showed a trend to be lower in cows with mastitis compared to healthy animals, but this difference was not significant (p < 0.10). IL-4 exerts an antagonistic action to IFN-γ and its main function is the regulation of IgE-mediated immune responses. In addition, IL-4 stimulates the differentiation of Th0 and Th2 lymphocytes, *i.e.*, the humoral immune response. Also, no difference in expression between both groups of animals was observed for the other genes. These findings are not conclusive since a similar pattern of expression of *IL-4* and *IL-10* would be expected because these interleukins produced by Th2 lymphocytes responsible for the humoral immune response. In addition, an expression pattern of *IL-10* opposite to that of the *IL-2*, *TNF-*αand *IFN-*γ genes would be expected, but this was only observed in the case of *IFN-*γ*.* Despite the non-significant difference in expression and the divergent results obtained for some genes, these findings suggest that animals with mastitis develop a preferentially cell-mediated immune response. Further studies are necessary to better characterize and understand the gene expression profiles in cows with mastitis, especially Gyr animals, since no studies of this type are yet available for Zebu cattle and, therefore, the results cannot be compared with other studies.

A significant difference in expression (p < 0.001) was observed when healthy cows of the two breeds were compared (Table 2), with the expression of the *IL-2* and *IFN-*γ genes being higher in BW Holstein cows. IL-2 induces the proliferation of activated mononuclear cells as well as of some activated epithelial cells. IL-2 can be suppressed by the expression of *IL-10* since an increase in IL-10 indirectly inhibits the differentiation of Th0 lymphocytes into Th1 lymphocytes. This result led to a reduction of IL-2 since this cytokine is mainly produced by Th1 lymphocytes (DeFranco *et al.*, 2007). IFN-γ is associated with the conversion of Th0 into Th1 lymphocytes and activation of macrophages and neutrophils, in addition to potentiating the action of TNF-α (Janeway *et al.*, 2002). Therefore, this cytokine is related to a Th1 immune response, *i.e.*, a cellular immune response. In addition, IFN-γ deficiency is associated with increased susceptibility to infections caused by intracellular microorganisms. Despite the significant difference in *IFN-*γ and *IL-2* expression, further studies are necessary to evaluate the expression profile in different breeds under different conditions.

IL-8, together with IL-1 and TNF-α, has been indicated as an important mediator of neutrophil recruitment to sites of inflammation. Some studies have shown that TNF-α and IL-8 are present in large amounts in milk from udders infected with Gram-negative bacteria such as *E. coli*, *K.pneumoniae* or *Pseudomonasaeruginosa*, but are detected at lower concentrations or are absent in milk of cows whose mammary gland is infected with *S. aureus*. In the present study, no significant difference in the expression of *TNF-*α or *IL-8* was observed when animals of the two breeds with mastitis were compared; however, expression of the two genes seemed to be higher in BW Holstein cows (Table 2), a fact that disagrees with the results obtained by others since in the present study all BW Holstein cows with mastitis were infected with *Staphylococcus* spp. and *K. pneumoniae* was the causative agent of mastitis in one Gyr cow (Shuster *et al.*, 1997; Riollet *et al.*, 2000; Bannerman *et al.*, 2004a, 2004b, 2005).

These divergent results might be explained by the fact that the immune response can differ according to bacterial strain and host, with the observation of wide individual variation. In agreement with the present findings, in an *in vitro* study Lahouassa *et al.* (2007) demonstrated that different *S. aureus* strains elicit different responses in epithelial cells of the mammary gland. In addition, in that study the intensity and level of expression of the genes analyzed (*IL-8*, *GRO-*α, *GRO-*β, *TNF-*α, *IL-1*β, *TGF-*β*1* and *IL-10*) varied according to the step of infection (3, 10 and 24 h after the addition of bacteria to the cell culture). According to those authors, these different responses imply in alternative routes of activation or at different signal transduction levels, reflecting what is observed *in vivo*. In the present study, the milk samples were collected immediately after the manifestation of clinical signs of mastitis, *i.e.*, it was not possible to determine when the animal was infected since two animals may manifest a disease on different days even when they were infected at the same date. In addition, there was no control of the type of strain that caused the infection and in some cases the causative agent of mastitis could not be identified (two Gyr cows infected with multiple agents). It should also be noted that the milk samples were collected from animals reared in commercial herds and, therefore, it was not possible to determine whether these animals were free of other infections or diseases, a fact that may have also influenced the present results.

For the genes studied here we do nopt know whether the expression levels reflect only messenger RNA or also protein levels, since factors of post-transcriptional regulation were not studied. Thus, it would be interesting to perform proteomic studies to confirm these results. In addition, analysis of the structure of the genes that were differentially expressed in this study, such as *IL-10*, would be useful to identify marker SNPs for mastitis resistance and susceptibility. Mastitis is a multifactorial disease which is influenced by numerous genes. Therefore, further studies including a larger number of genes and animals in different steps of infection are necessary to better understand the immune response mechanism and to develop more efficient strategies for the control and eradication of this disease. In addition, studies using experimental populations, including the history of mastitis, somatic cell count of each animal, calving order and lactation stage, as well as studies inoculating specific bacterial strains and collecting samples at different times after inoculation, would greatly contribute to the understanding of the physiopathology of this disease.

It should be emphasized that the *IL-4*, *IL-6*, *IL-8* and *TNF-*α genes were not differentially expressed in any of the conditions studied (p > 0.05), demonstrating that these genes are not good markers or indicators of mastitis under the analyzed conditions. Further experiments will be performed to investigate not only the genes studied here, but also other genes that might be involved in mastitis resistance, especially in Zebu animals, since most studies so far have been conducted on breeds of European origin. These studies may contribute to a better understanding of the immune responses that occur in mastitis, a disease that significantly affects milk quality and yield especially in Zebu breeds which represent the basis of the cattle-raising industry in Brazil.

## Figures and Tables

**Table 1:** Primer pairs and optimized conditions used for determining bovine gene expression by quantitative real-time PCR

Gene	Oligonucleotides (5'-3') F: forward; R: reverse	Reference	Primer (nM)	cDNA (ng/μL)	DT (°C)
*IL-2*	F: GGATTTACAGTTGCTTTTGGAGAAA R: GCACTTCCTCTAGAAGTTTGAGTTCTT	1	400	400	65.4
*IL-4*	F: CATGCATGGAGCTGCCTGTA R: AATTCCAACCCTGCAGAAGGT	2	400	200	79.3
*IL-6*	F: TCAGCTTATTTTCTGCCAGTCTCT R: TCATTAAGCACATCGTCGACAAA	1	400	400	72.6
*IL-8*	F: CACTGTGAAAAATTCAGAAATCATTGTTA R: CTTCACCAAATACCTGCACAACCTTC	1	400	100	77.2
*IL-10*	F: CCAAGCCTTGTCGGAAATGA R: GTTCACGTGCTCCTTGATGTCA	3	200	100	80.2
*TNF-*α	F: TCTTCTCAAGCCTCAAGTAACAAGT R: CCATGAGGGCATTGGCATAC	1	400	100	83.0
*IFN-*γ	F: TGGATATCATCAAGCAAGACATGTT R: ACGTCATTCATCACTTTCATGAGTTC	1	400	200	87.5
*GAPDH*	F: GGCGTGAACCACGAGAAGTATAA R: CCCTCCACGATGCCAAAGT	1	400	100	82.6

References: ^1^Leutenegger *et al.*, 2000; ^2^Waldvogel *et al.* 2000; ^3^Moussay *et al.*, 2006.

**Table 2:** Relative gene expression in animals with mastitis compared to animals without mastitis and the respective standard errors of the mean.

Gene	*IL-2*	*TNF*-α	*IL-8*	*IL-10*	*IL-4*	*IFN*-γ	*IL-6*
Animals of both breeds	3.55	-2.43	2.05	20.71*	-3.85	1.26	3.90
	±8.33	±1.10	±5.26	±61.33	±0.92	±3.09	±9.60

BW Holstein cows	-1.92	-5.07	1.70	35.66***	-1.80	-1.44	1.46
	±1.71	±0.78	±6.25	±162.70	±3.01	±2.47	±4.42

Gyr cows	24.23	-1.16	2.47	12.03^+^	-8.27^+^	2.29	10.41
	±85.82	±3.44	±10.03	±53.69	±0.64	±8.72	±43.21

Gyr and BW Holstein cows without mastitis	-43.87***	-4.40	-4.85	-2.48	-10.98	-38.57***	-5.89
	±0.07	±0.85	±0.76	±1.72	±0.45	±0.09	±0.52

Gyr and BW Holstein cows with mastitis	1.06	-1.01	-3.34	-7.35^+^	-50.59	-11.70	1.21
	±3.83	±4.14	±1.22	±0.65	±0.11	±0.34	±4.95

Positive numbers: higher expression in animals with mastitis; negative numbers: lower expression in animals with mastitis (*p; ***p; ^+^p).
